# Structure and Mutation of the Native Amine Dehydrogenase MATOUAmDH2

**DOI:** 10.1002/cbic.202200136

**Published:** 2022-04-07

**Authors:** Megan Bennett, Laurine Ducrot, Carine Vergne‐Vaxelaire, Gideon Grogan

**Affiliations:** ^1^ Department of Chemistry University of York Heslington York YO10 5DD UK; ^2^ Génomique Métabolique, Genoscope Institut François Jacob, CEA, CNRS Univ Evry Université Paris-Saclay 91057 Evry France

**Keywords:** amines, amine dehydrogenases, biocatalysis, NADPH, oxidoreductases

## Abstract

Native amine dehydrogenases (nat‐AmDHs) have recently emerged as a potentially valuable new reservoir of enzymes for the sustainable and selective synthesis of chiral amines, catalyzing the NAD(P)H‐dependent ammoniation of carbonyl compounds with high activity and selectivity. MATOUAmDH2, recently identified from the Marine Atlas of Tara Oceans Unigenes (MATOUv1) database of eukaryotic genes, displays exceptional catalytic performance against its best identified substrate, isobutyraldehyde, as well as having broader substrate scope than other nat‐AmDHs. In the interests of providing a platform for the rational engineering of this and other nat‐AmDHs, we have determined the structure of MATOUAmDH2 in complex with NADP^+^ and also with the cofactor and cyclohexylamine. Monomers within the structure are representative of more open and closed conformations of the enzyme and illustrate the profound changes undergone by nat‐AmDHs during the catalytic cycle. An alanine screen of active site residues revealed that M215A and L180A are more active than the wild‐type enzyme for the amination of cyclohexanone with ammonia and methylamine respectively; the latter suggests that AmDHs have the potential to be engineered for the improved production of secondary amines.

## Graphical abstract

**The native amine dehydrogenase** MATOUAmDH2, catalyzes the ammoniation of small aliphatic ketones with high selectivity. Structural analysis reveals domain movements associated with the formation of the active site. This informed the design of mutants that have superior activity for the amination of cyclohexanone with either ammonia or methylamine.

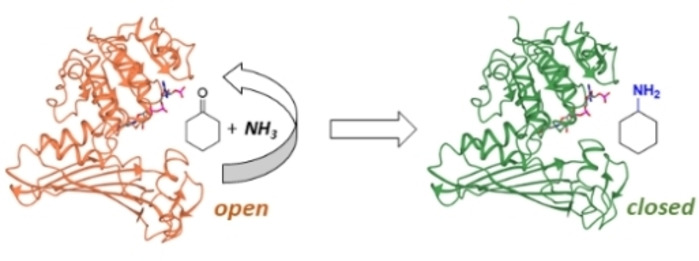

## Introduction

The synthesis of chiral amines in optically enriched form through reductive amination is a significant process in industrial synthetic organic chemistry as it produces valuable chiral intermediates and pharmaceutical molecules.^[1]^ Biocatalysts have emerged as an attractive and competitive option for the synthesis of chiral amines owing to the sustainable chemistry credentials of enzymatic processes, which reduce the need for toxic reagents, rare precious metals and hazardous reaction conditions, but also confer excellent enantioselectivities upon amine synthesis reactions.[^2^, ^3^] The direct conversion of readily available prochiral carbonyl compounds to amines is one especially valuable type of enzymatic reaction. This can be catalyzed by transaminases,[^4^, ^5^] at the expense of an ammonia donor and the cofactor PLP, but also by NAD(P)H‐dependent oxidoreductases[^6^, ^7^, ^8^, ^9^, ^10^] that can, in some cases, catalyze the two‐step conversion of carbonyls to amines through active, sequential catalysis of prochiral imine formation and imine reduction, when presented with carbonyl and amine substrates. The major classes of enzymes capable of this transformation include: Reductive Aminases (RedAms),[^11^, ^12^] a subclass of imine reductases (IREDs)[^13^, ^14^] that catalyze the formation of amines from, particularly, cyclic ketones and small amines when provided in an equimolar ratio; amino acid dehydrogenases (AADHs),[^15^, ^16^] which catalyze the reductive amination of amino acids with ammonia but can be altered to accept non‐carboxylated ketone substrates through engineering of their actives, giving rise to amine dehydrogenases (AmDHs).[^17^, ^18^, ^19^, ^20^, ^21^, ^22^, ^23^, ^24^] In recent examples, such engineered AmDHs have been applied to the synthesis of, for example, (*R*)‐1,3‐dimethylbutylamine, (*R*)‐amphetamine and (*R*)‐1‐phenylethylamine from suitable carbonyl precursors using enzyme variants based upon leucine (LeuDHs)^[17]^ phenylalanine dehydrogenase (PheDHs)^[18]^ and ϵ‐deaminating L‐lysine dehydrogenase^[25]^ respectively.

**Scheme 1:**
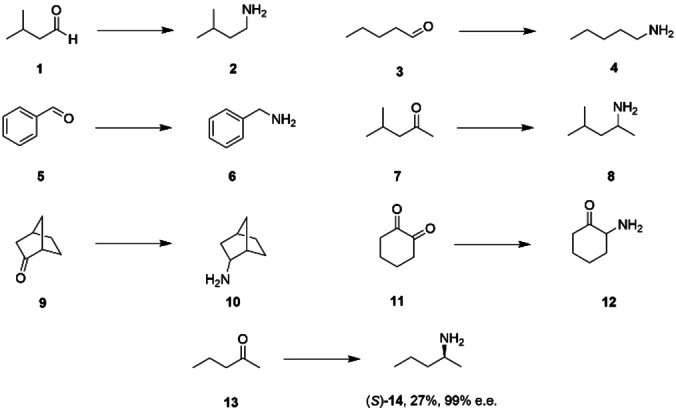
Carbonyl substrates transformed to amines by MATOUAmDH2 with the addition of NH_3_ and NADPH.[^29^, ^30^]

More recently however, native amine dehydrogenases (nat‐AmDHs) have been discovered that naturally catalyze the amination of ‘neutral’ carbonyl compounds using ammonia. The first nat‐AmDHs described were those, such as ‘AmDH4’ from *Petrotoga mobilis*,^[26]^ that aminated a carbonyl group remote from a carboxylic acid, converting levulinic acid (4‐oxopentanoic acid) to (4*S*)‐4‐aminopentanoic acid. AmDH4 was subsequently engineered to convert 2‐pentanone to (2*R*)‐aminopentane by mutating the carboxylate binding site.^[27]^ However, further searches of the genomic resource using the AmDH4 sequence as a model, and also its structure as a guide to suggesting new specificity determinants within their active sites, led to the description of further nat‐AmDHs with a specificity for neutral carbonyl compounds.^[27]^ *Cfus*AmDH from *Cystobacter fuscus* and *Msme*AmDH from *Mycobacterium smegmatis* converted different aliphatic ketones and aldehydes such as cyclohexanone and isobutyraldehyde to cyclohexylamine and isobutylamine, respectively. Subsequent searches of large metagenomics databases, including the Marine Atlas of Tara Oceans Unigenes (MATOUv1), comprising eukaryote unigenes,^[28]^ revealed several more AmDH sequences that were evaluated for activity by Caparco and co‐workers after sequence design and heterologous expression in *E. coli*.^[29]^ Of these, the enzyme designated MATOUAmDH2 displayed distinctive properties. With 33 % sequence identity shared between this and both *Cfus*AmDH and *Msme*AmDH, MATOUAmDH2 displayed a 71: 1 preference for NADPH over NADH, a specific activity at 50 °C of 11 U mg^−^1 for the amination of the substrate isobutyraldehyde **1** with ammonia, but also measurable activity for pentaldehyde **3** and benzaldehyde **5** (Scheme 1). MATOUAmDH2 was also unique among the target enzymes in displaying activity toward methyl isobutyl ketone (MIBK) **7**, norcamphor **9** and the dicarbonyl cyclohexane‐1,2‐dione **11**, transforming this to the monoamine product **12**, although the enantiomeric excess of the 2‐amino cyclohexanone was not determined.

Relative activities of 6 % and 16 % for cyclohexanone with ethylamine and methylamine respectively (*vs* cyclohexanone with ammonia) were also reported, hinting at a potential for the synthesis of secondary amines by MATOUAmDH2. Further studies established that MATOUAmDH2 also catalyzed the enantioselective production of (*S*)‐short alkylamine and alkylaminoalcohols from suitable precursors including 2‐pentanone **13**, which was converted to (2*S*)‐2‐pentanamine **14** with 27 % conversion and 99 % e.e.^[30]^ The broader and distinctive substrate specificity of MATOUAmDH2 identifies it as an interesting candidate for *in vitro* evolution strategies targeted at improving or altering its activity, selectivity and process suitability. In this report we present a structural and mutational analysis of MATOUAmDH2, in the interests of providing a robust platform for rational engineering of this nat‐AmDH.

## Results and Discussion

### Expression, purification and crystallization

The gene encoding MATOUAmDH2^[29]^ was expressed in *E. coli* pET22b and the enzyme purified using nickel affinity chromatography and gel filtration using protocols described in the Supporting Information (SI Section 1). The enzyme was crystallized (SI Section 4 and 5) and the data collected on the best crystals were solved and refined to a resolution of 2.34 Å. Further attempts to obtain a ligand complex using this construct were unsuccessful, so the MATOUAmDH2 gene was subcloned into the pETYSBL‐LIC3C vector^[31]^ and expressed again (SI Section 2 and 3), this time yielding pure protein which was crystallized in the presence of both NADP^+^ and cyclohexylamine (CHA) (SI Section 4 and 5). The resulting data were refined to a resolution of 2.08 Å and the experimental maps clearly showed density corresponding to CHA in the active site, as described below. Omit maps showing the cofactor density are given in Figure S3 (SI Section 5). Data collection and refinement statistics for both structures are given in Table S2.

### Structure of MATOUAmDH2

**Figure 1:**
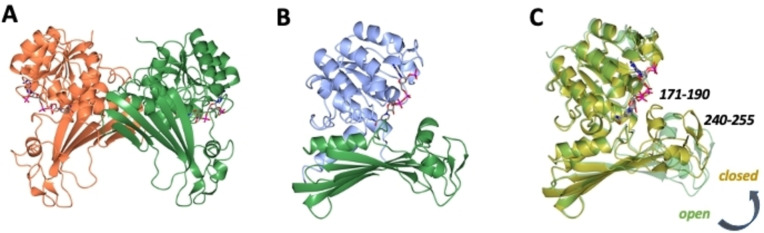
A: MATOUAmDH2 dimer with subunits shown in green and coral. B: MATOUAmDH2 monomer with N‐terminal Rossman domain (residues 8–158; 309–351) shown in blue and sheet domain (residues 159–309) shown in green. NADP^+^ is shown in cylinder format with carbon atoms in grey. C: Superposition of MATOUAmDH2 closed and open monomer conformation shown in gold and green respectively. The closure of loops 171–190 and 240–255 over the active site is indicated.

The first structure of the amine dehydrogenase MATOUAmDH2 was determined in complex with NADP^+^ to a resolution of 2.32 Å. The crystals were obtained in space group *P*2_1_2_1_2_1_ and featured four molecules in the asymmetric unit, comprising two dimers (Figure 1A) and permitting observation of open and closed conformers of the enzyme subunits. Electron density for the backbone and side chains could be modelled for most regions save for residues 189–193 and 190–193 in monomers C and D respectively. The two‐domain structure of the MATOU monomer is similar to that observed with *Cfus*AmDH and *Msme*AmDH, with a largely N‐terminal Rossman fold domain (residues 8–158; 309–351) and a largely C‐terminal domain 159–309 comprising a seven‐stranded beta sheet (Figure 1B). Analysis by the DALI server^[32]^ revealed that the closest structural homologs were indeed the *Cfus*AmDH from *Cystobacter fuscus* (PDB code 6IAU; 33 % sequence identity; rmsd 2.4 Å over 340 Cα atoms) and *Msme*AmDH (PDB code 6IAQ; 33 %; rmsd 2.4 Å over 340 Cα atoms) previously presented by our groups. The cofactor NADP^+^ was bound within the cleft between the two domains in each monomer.

The monomers that constitute each dimer (A/D and B/C) in the asymmetric unit presented two distinct conformations: a more open form (subunits B and D), and a much more closed form (A and C), where loops had closed over the active site containing the NADP^+^. Open and closed forms of AmDH monomers were also observed in a single structure of the amine dehydrogenase AmDH4 from *Petrotoga mobilis* (6G1 M)^[27]^ although that enzyme is different from MATOUAmDH2 in binding and transforming keto acid substrates.^[26]^ The closed conformations in subunit B and D of MATOUAmDH2 were similar to the conformations observed for *Cfus*AmDH (6IAU) and *Msme*AmDH (6IAQ) obtained previously by our group. The closed monomers show that the major contributions to this closure are the loops in the beta sheet domain, most especially those between residues P240 and G255 and Y171 and E190 (Figure 1C). The loop closure serves to form the active site and to bring many amino acid residues within closer proximity to bound ligands, as described below.

### Active site of MATOUAmDH2

**Figure 2:**
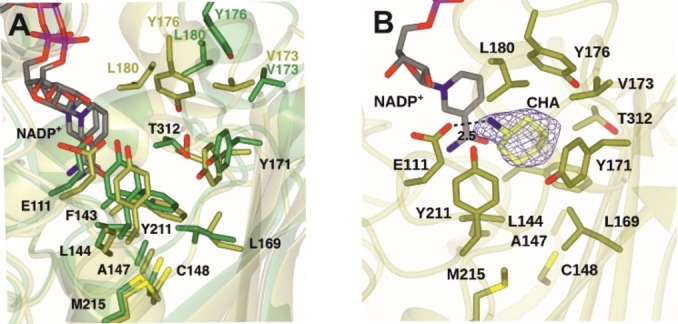
A: Superposition of active sites of MATOUAmDH2 when in the open and closed conformations, with side chain carbon atoms in green and gold respectively. Where significant movement of residues has occurred, as a result of the relocation of loop 171–190 upon closure, the side chains have been labelled in green and gold for open and closed positions; B: Active site of MATOUAmDH2 in complex with NADP^+^ and cyclohexylamine (CHA). Electron density corresponds to the omit (*F*o – *Fc*) map at a level of 3*σ* observed prior to ligand building and refinement. Distance is given in Ångstroms.

The active site of MATOUAmDH2 approximates in shape to a rectangular box (Figure 2A). One end side presents the catalytic residue, E111, which is thought to activate ammonia for attack at the carbonyl group of the substrate in the cavity. Mutation of the equivalent E102 into alanine in AmDH4 from *P. mobilis* greatly reduced the affinity for ammonia in that mutant, which was almost inactive.^[27]^ The two long sides feature the pyridinium ring of NADP^+^, and on the other side, tyrosine residue 171, which would each stack against the substrate. The floor of the box features hydrophobic residues L144 and L169, F143, A147 and M215, but also C148. The fourth, end side is formed by T312 and Y176, V173 and L180 from the mobile loop Y171–E190, which also provides the ceiling. A superimposition of the open and closed forms (Figure 2A) shows that Y176 and L180 have moved into position as a result of the loop movement and consequent active site closure (Figure 2A). Following subcloning and expression using pETYSBLIC‐3 C, which equips MATOUAmDH2 with a 12 amino acid extension leading to the hexahistidine tag at the N‐terminus, further crystals, formed in the presence of both NADP^+^ and the reaction product cyclohexylamine, were obtained, with an improved resolution of 2.08 Å. In this case the crystals were obtained in space group *P*3_2_1 with one molecule in the asymmetric unit, which was in the closed conformation equivalent to that observed with the C monomer of the NADP^+^‐only complex (rmsd of 0.40 Å over 340 Cα atoms). In this structure, the omit maps revealed clear electron density adjacent to E111, which was modelled and refined successfully as cyclohexylamine (Figure 2B). The ligand was, as expected, positioned with its ring plane sandwiched between the nicotinamide ring of NADP^+^ and the side chain of Y171 and with its nitrogen atom 2.5 Ångstroms from the OE2 carboxylate atom of E111.

### MATOUAmDH2 has a larger active site than CfusAmDH

**Figure 3:**
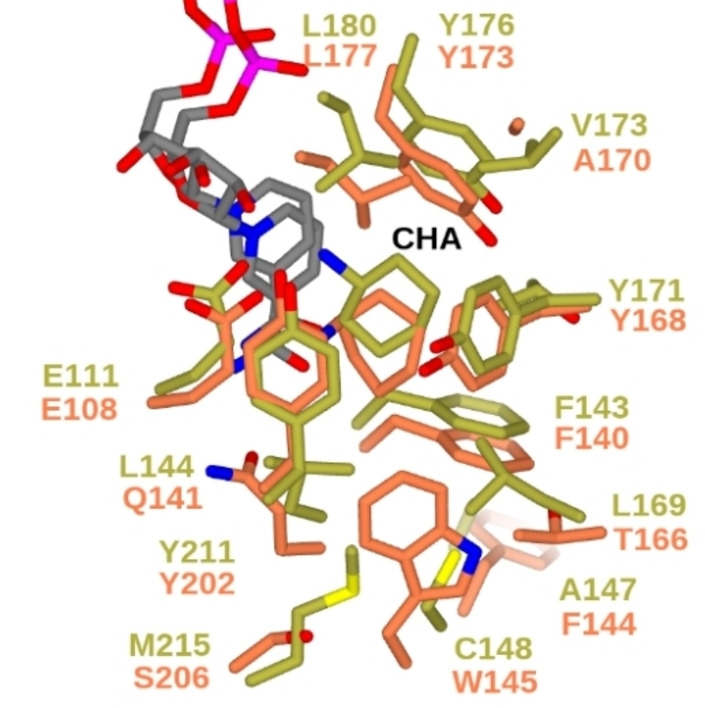
Superposition of active sites of MATOUAmDH2 closed conformation in complex with cyclohexylamine (**CHA**) (gold) and that of *Cfus*AmDH (PDB code 6IAU; coral), also in complex with **CHA**, showing conservation of residues in the ‘ceiling’ of the active site but many differences at the ‘floor’.

The structure of the active site of MATOUAmDH2 can be compared to that of *Cfus*AmDH,^[27]^ in an effort to shed light on the broader substrate scope and superior activity of the first enzyme. Conserved residues include the catalytic glutamate E111 (E108 in *Cfus*AmDH), Y211 (Y202), Y171 (Y168), Y176 (Y173), F143 (F140) and L180 (L177) (Figure 3). The most notable differences in the active site between MATOUAmDH2 and *Cfus*AmDH are the substitutions in the floor of the active site. First, A147 (F144) and C148 (W145) may provide substantially increased room to accommodate bulkier substrates. The other substitutions replace hydrophilic side chains with hydrophobic ones: L169 for T166, L144 for Q141 and M215 for S206; the presence of these residues increases the hydrophobic character of the active site pocket. The recently published results regarding activity toward hydroxy ketones are in line with this feature.^[21]^ Lower production of 1‐methoxypropan‐2‐amine, 3‐aminobutan‐1‐ol and 2‐aminobutan‐1‐ol were obtained with MATOUAmDH2 compared to *Cfus*AmDH.

The ligand cyclohexylamine (CHA) is also bound in a different orientation, with the cyclohexyl ring more in plane with the side chain of Y171 and the nicotinamide ring of NADP^+^ (Figure 3).

### Cofactor preference of MATOUAmDH2

**Figure 4:**
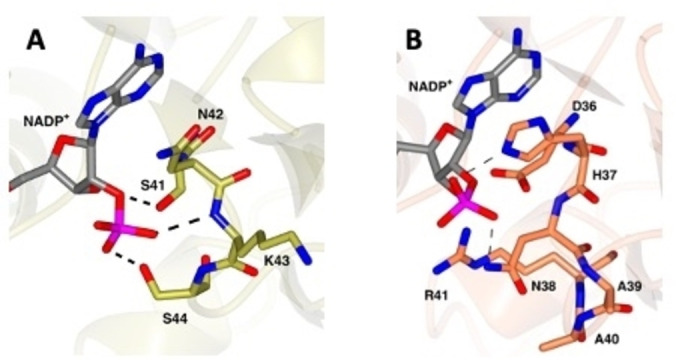
A: NADP^+^ binding loop in MATOUAmDH2. The phosphate of NADP^+^ makes close contacts with the side chains of S41 and S44, and the backbone NH of N42. B: NADP^+^ binding loop in *Cfus*AmDH. The phosphate of NADP^+^ makes close contacts only with the side chains of H37 and N38.

Away from the active site, there are notable differences between MATOUAmDH2 and *Cfus*AmDH in the residues that bind the ribose 2’ phosphate of NADP^+^, and which may govern nicotinamide cofactor specificity in the enzymes. In MATOUAmDH2 the phosphate is bound by the side chains of S41 and S44 and the backbone N−H of K43 that are found in the phosphate binding loop (Figure 4A); the corresponding residues in *Cfus*AmDH are D36 and N38 although the former does not interact with the phosphate.

The adenine ring of the cofactor stacks against N42 in MATOUAmDH2, but H37 in *Cfus*AmDH, which also provides coordination to the phosphate (Figure 4B). The overall effect in MATOUAmDH2 is to restrict the movement of the phosphate with more binding interactions and possibly explaining the stricter specificity of MATOUAmDH2 for the phosphorylated cofactor over NADH (71: 1) described previously^[30]^ compared to *Cfus*AmDH, which displays a preference of 2.67 : 1 in catalytic efficiency for NADPH over NADH.^[29]^

### Mutational analysis of MATOUAmDH2

In the interests of investigating the structural determinants of broader substrate specificity and superior activity in MATOUAmDH2, a number of active site mutations were made (SI Section 6) and the performance of the WT and mutants compared using kinetic measurements (Table 1 and SI Section 7). Mutations F143A, L144A, L169A and M215A were designed to probe hydrophobic interactions on the floor of the active site; L180A and T312A were constructed to examine the effect of mutation on these residues that are brought into proximity with the ligand through loop closure.

**Table 1:** Kinetic constants determined for WT MATOUAmDH2 and active site mutants for cyclohexanone as substrate with 2 M ammonium formate buffer at pH 8.0.

Variant	*k*_cat_ [s^−1^]	*K*_M_ (mM)	*k*_cat_/*K*_M_ [s^−1^ mM^−1^]
WT	0.082	0.40 ±0.07	0.21
L180 A	0.033	0.61 ±0.12	0.05
T312 A	0.038	2.31 ±0.29	0.02
M215 A	0.063	0.23 ±0.03	0.27

**Figure 5:**
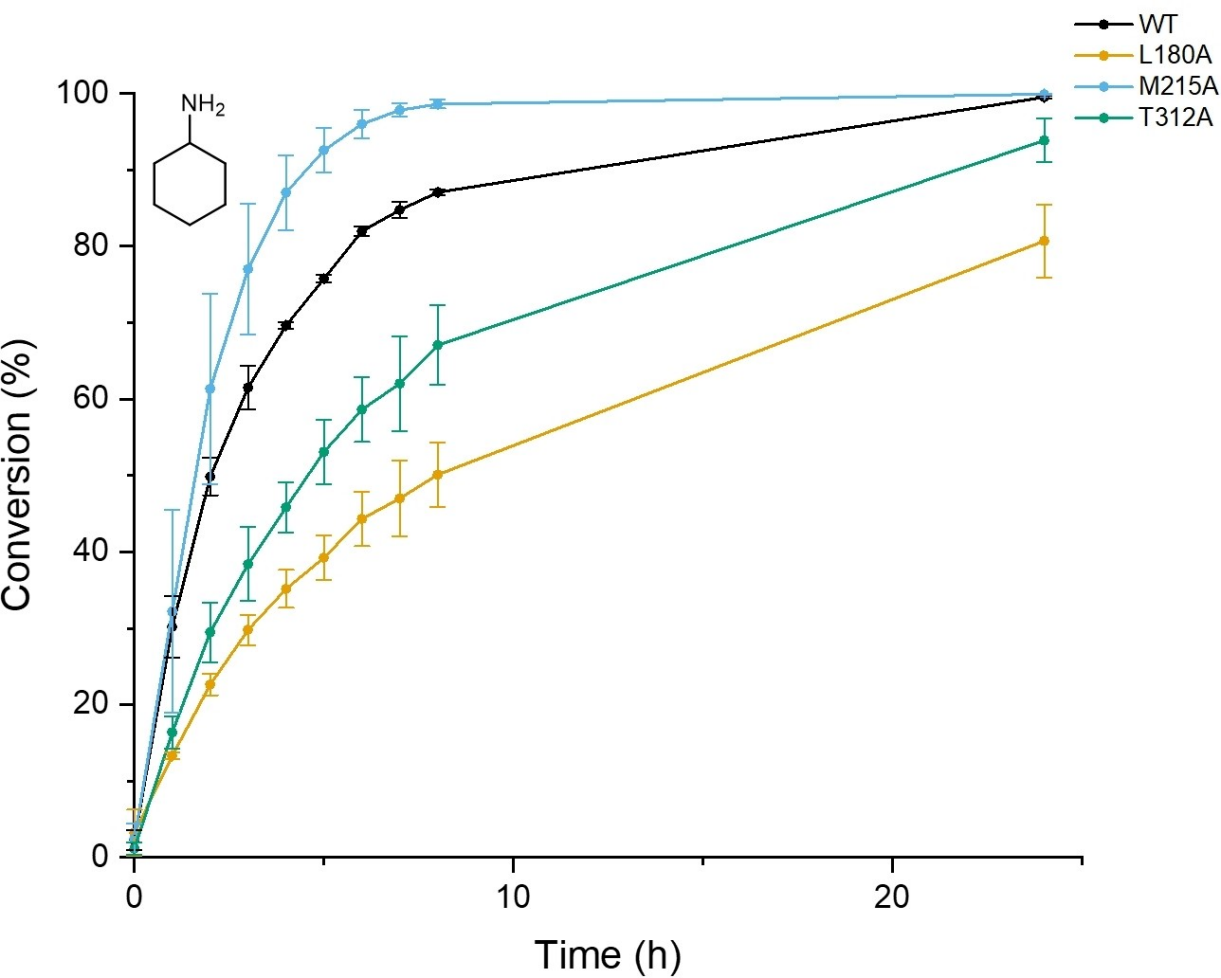
Biotransformation of cyclohexanone with ammonia by MATOUAmDH2 and mutants.

Muteins were expressed and purified as for the WT enzyme. Interestingly, mutants F143A, L144A and L169A precipitated very soon after purification and could not be used for assay, indicative of roles in stabilization of the enzyme. Mutant L180A displayed slightly higher *K*_M_ values than the WT, but the T312A mutant displayed a more profound change with a near 6‐fold increase in *K*_M_, indicative of an important role for this side chain in substrate binding. This might be further explored through saturation mutagenesis with a specific focus on more hindered substrates such as substituted cyclohexanones. For M215A, *K*_M_ was decreased by a factor of 1.73, and *k*_cat_/*K*_M_ improved from 0.21 to 0.27 s^−1^mM^−1^, indicating that there may be considerable potential for improving AmDH activity through engineering at this locus at the floor of the active site. This improvement in the activity of M215A was confirmed by biotransformation experiments (Figure 5, SI Section 8) that showed this mutant catalyzed the amination of cyclohexanone with ammonia at a superior rate than the wild‐type enzyme.

Further biotransformations with mutants studied their performance with respect to the amination of cyclohexanone using a 25‐fold excess of methylamine as the amine donor. Interestingly in this case the M215A mutant only performed as well as the wild‐type, but both the T312A, and especially the L180A mutants, displayed significantly improved activity, with 41 % conversion over 24 h in the latter case vs only 8 % for the wild‐type (Figure S5). The structure of MATOUAmDH2 in complex with cyclohexylamine (Figure 3) suggests that the improved activity for the production of *N*‐methyl cyclohexylamine may be due to the increased space afforded by the L−A mutation in the roof of the active site, permitting improved binding of the relevant alkyl imine intermediate from solution for reduction. Although the performance of MATOUAmDH2 mutants in this regard does not match the abilities of Reductive Aminases to catalyze the production of secondary amines from ketone and amine precursors supplied at a 1 : 1 ratio,^[11]^ it nevertheless suggests a broader scope of activity in AmDHs that may be explored through further mutation and the identification of further enzymes from sequence databases.

### Modelling

**Scheme 2:**
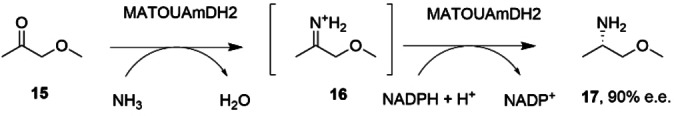
Reductive amination of 1‐methoxypropanone **13** to (*S)‐*MOIPA **15** using MATOUAmDH2.^[30]^

The acquisition of the MATOUAmDH2 structure in complex with cyclohexylamine permitted us to model substrates into the active site in order to rationalize the observed stereoselectivity of the enzyme. For example, MATOUAmDH2 was shown to catalyze the amination of 1‐methoxypropan‐2‐one **15** to (*S*)‐1‐methoxypropan‐2‐amine **17** (MOIPA) (Scheme 2), an important chiral element of herbicide molecules including (*S*)‐metolachlor and (*S*)‐dimethenamid,^[33]^ with 90 % e.e.^[30]^

**Figure 6:**
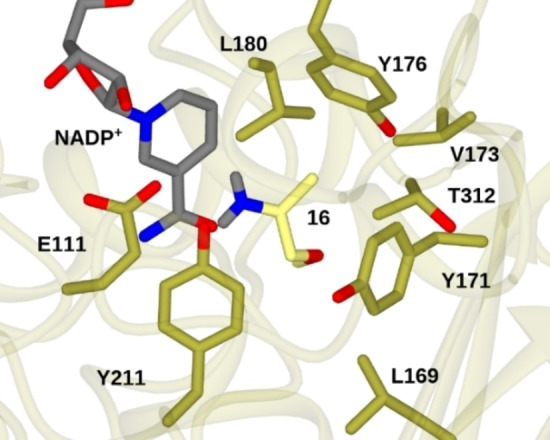
Model of imine **16** bound in the active site of MATOUAmDH2 calculated using Autodock VINA.^[34]^

In doing so, we have used Autodock Vina^[34]^ to model the intermediate iminium **16** into the newly described active site of MATOUAmDH2 (Figure 6, SI Section 9).

The top pose suggested that **16** would be bound in an orientation such that hydride would be delivered to the *pro*‐*R* face of the iminum to deliver the (*S*)‐enantiomer product as observed, with the longer substituent pointing downwards into the active site pocket. Intriguingly, this pose also suggests that larger substrates may indeed be accommodated within this pocket.

## Conclusion

Amine dehydrogenases are an emerging class of enzymes that have great potential for application in industrial biocatalysis as tools for the asymmetric synthesis of primary amines. The structure of MATOUAmDH2 reveals the basis for extended substrate scope in this enzyme, and has informed the creation of mutants that are superior in one case in activity, and in the other in improved recognition of imine intermediates that lead to secondary amines. These studies will enable further protein engineering experiments to be performed in an effort to broaden specificity and improve both stability and activity for process applications.

## Conflict of interest

The authors declare no conflict of interest.

1

## Supporting information

As a service to our authors and readers, this journal provides supporting information supplied by the authors. Such materials are peer reviewed and may be re‐organized for online delivery, but are not copy‐edited or typeset. Technical support issues arising from supporting information (other than missing files) should be addressed to the authors.

Supporting Information

## Data Availability

The data that support the findings of this study are available from the corresponding author upon reasonable request.
