# Supplementary material for: Structure and Mutation of the Native Amine Dehydrogenase MATOUAmDH2
Source: Chembiochem. 2022 Apr 7;23(10):e202200136. doi: 10.1002/cbic.202200136 (PMC9325545; doi:10.1002/cbic.202200136)
Supplement: Supplementary file 1 — Supporting Information [file CBIC-23-0-s001.pdf]

ChemBioChem

Supporting Information

Structure and Mutation of the Native Amine Dehydrogenase MATOUAmDH2

Megan Bennett, Laurine Ducrot, Carine Vergne-Vaxelaire, and Gideon Grogan*

1. Expression and Purification of MATOUAmDH2 from pET22b

The MATOUAmDH2 gene had been cloned into a pET22b vector with a non-cleavable N-terminal histidine tag as described previously.[1] The recombinant plasmid was used to transform BL21(DE3) (New England BioLabs) cells and heat shocked at 42ºC for 45 s. 1 mL of Luria Bertani (LB) medium was added and the suspension was incubated at 37ºC for 1 h with shaking at 120 r.p.m. 100 µL of this culture were then added to LB agar plates containing 100 µg mL^{-1} ampicillin and these were incubated at 37ºC overnight. Single colonies were selected and inoculated into 10 mL LB broth containing 100 µg mL^{-1} ampicillin. These cultures were incubated for 18 h at 120-180 r.p.m at 37ºC with shaking and then transferred to 2 L flasks containing 1 L LB broth containing 100 µg mL^{-1} ampicillin. These cultures were incubated at 37ºC until they reached an absorbance value of 0.6 at 600 nm, upon which they were induced with the addition of 1 mM isopropyl-β-D-thiogalactopyranoside (IPTG). Following induction, cells were left to grow at 16ºC for 18 h with shaking at 180 r.p.m. Cells were then harvested using a Lynx6000 centrifuge (Thermo Scientific) at 9760 *g* for 20 min. The pellets were resuspended in 100 mL of a buffer containing 50 mM Tris-HCl, 300 mM NaCl and 20 mM imidazole at a pH of 7.1. Resuspended cells were disrupted using a cell disruptor homogenizer (Constant Systems Ltd) at a pressure of 26-27 kPsi. Lysed cells were then centrifuged at 38,571 *g* for 40 min and the supernatant retained for enzyme purification.

The cell supernatant was filtered through a 0.45 µm filter before being loaded onto a 5 mL FF HisTrap column (GE Healthcare) pre-loaded with nickel sulfate. MATOUAmDH2 was eluted using a low UV imidazole gradient formed from start and limit buffers of 20 mM to 500 mM respectively, each contained within a buffer of 50 mM Tris-HCl and 300 mM NaCl at pH 7.1. Fractions containing MATOUAmDH2, as determined by SDS-PAGE were concentrated to a volume of 2 mL using 10,000 MWCO concentrators before being filtered, as above, and loaded onto a Superdex gel filtration S200 16/60 column. Following elution, fractions were analysed using SDS-PAGE (**Figure S1**).

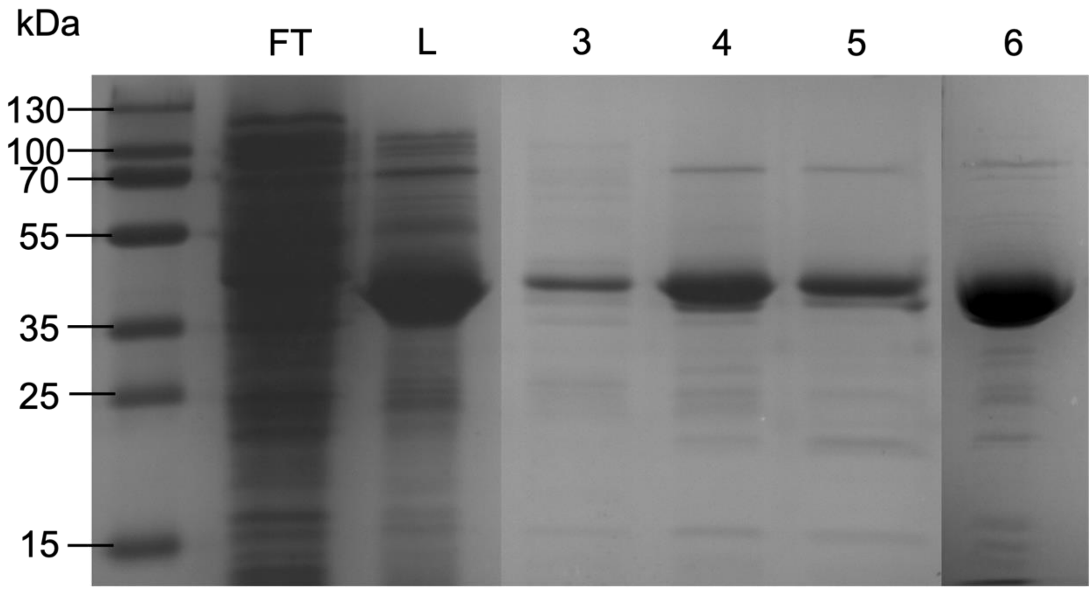

Figure S1: SDS-PAGE gel of purification fractions for the MATOUAmDH2 pET22b construct. 12% SDS-PAGE gel showing Ni^{2+} affinity and gel filtration fraction(s). The broad range ladder was from ThermoFisher and reference molecular masses are displayed in kDa. Labels are as follows: **FT**: 10x diluted flow through from the nickel column after loading; **L**: 10x diluted sample which was loaded onto the nickel column; Lane **3**, **4** and **5**: 10x diluted fractions from nickel column AKTA run as determined from chromatographs; Lane 6: 20x diluted purified and combined fractions after gel filtration.

2. Subcloning of MATOUAmDH2 gene in pETYSBLIC-3C

The gene encoding MATOUAmDH2 was subcloned into the pETYSBLIC-3C vector in order to explore an alternative construct for protein crystallization. The MATOUAmDH2 gene was amplified from the pET22b construct by PCR using the primers in **Table S1**.

Table S1. PCR primers for subcloning MATOUAmDH2 into pETYSBLIC-3C.

**Primers**	
Forward	TTCCAGGGACCAGCATCGCCTCTGCGTGTTGCG
Reverse	GGAGAAGGCGCCTTTAAAGACGGCCATGAACGTACGC

Following the PCR, 20 units Dpn1 (1 µL) were added to the mixture, which was then made up to 50 µL with water and CutSmart buffer (NEB). The Dpn1 digestion reaction was incubated at 37ºC for 20 min and then a heat inactivation step was performed at

80ºC for 20 min. The reaction was run on an agarose gel and the band of a size corresponding to the MATOUAmDH2 gene (approx 1 kB) excised and the DNA purified. In-Fusion cloning (Takara Bio) was carried out using the protocol provided by the vendor using a 2:1 ratio of insert to vector. The In-Fusion reaction was incubated at 50ºC for 15 min and then placed on ice. 2.5 µL of the reaction was then added to 25 µL of thawed Stellar competent cells and placed on ice for 30 min. The cells were then heat shocked at 42ºC for 45 s and then the transformed cells placed on ice for 2 min. The total volume was brought to 500 µL with SOC media and shaken at 180 r.p.m at 37ºC for 1 h. The culture was plated out onto agar containing kanamycin (30 µg mL^{-1}) and incubated at 37ºC overnight. Single colonies were selected and plasmids miniprepped. The successful cloning was first confirmed by a double restriction digest using NcoI and NdeI and then by DNA sequencing.

3. Expression and Purification of MATOUAmDH2 from pETYSBLIC-3C

MATOUAmDH2 in the PETYSBLIC3C vector was produced in an identical manner to that from the construct in pET22b, except that the antibiotic marker in transformation experiments and cell growths was kanamycin, rather than ampicillin, and was added in all cases to a final concentration of 30 µg mL^{-1}. Gel fractions, run as above, for the MATOUAmDH2 pETYSBLIC-3C construct are provided below in **Figure S2**.

4. Crystallization

For MATOU AmDH2 purified from pET22b expression

Fractions containing MATOUAmDH2, as determined by SDS-PAGE were pooled and concentrated to 10 mg mL^{-1} for crystallization experiments. Purified MATOUAmDH2 was complexed with 10 mM $NADP^+$ and subjected to crystallization trials using a range of commercially available screens in 96-well-plate sitting-drop format using a Mosquito robot (TTP LabTech). For crystallization drops a 1:1 ratio of protein solution (150 nL) to mother liquor (150 nL) was used. After initial trials, the best hits were obtained from conditions containing 0.2 M $(NH_4)_2SO_4$, 0.1 M Bis-Tris pH 6.0 and 20% (w/v) PEG 3350. Crystals were optimised in 48-well plate format using 800 nL plus 800 nL (1:1)

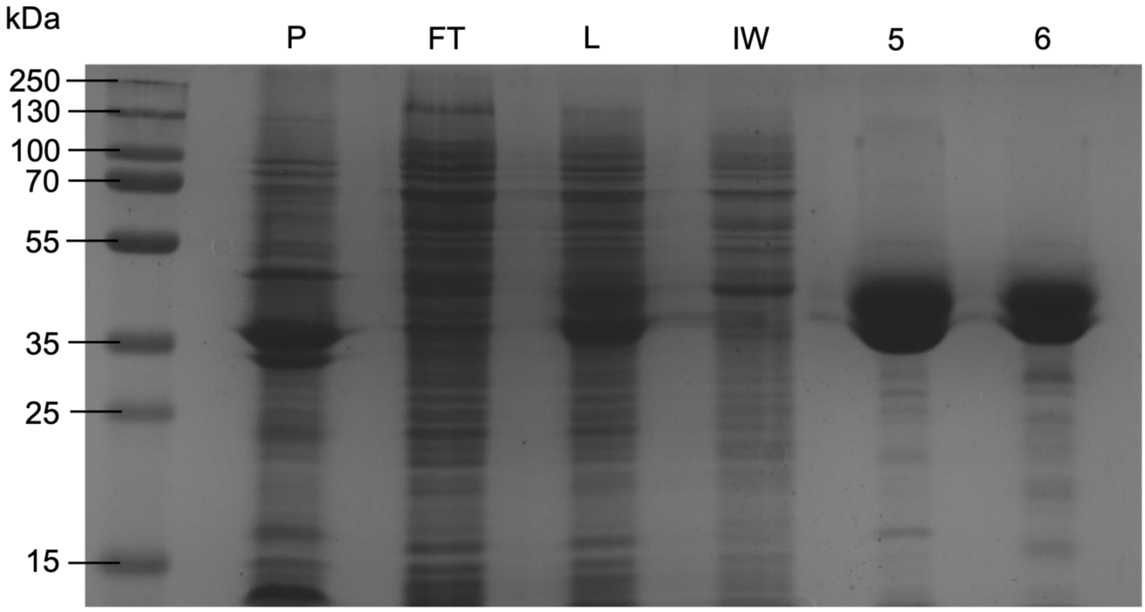

Figure S2: SDS-PAGE gel showing Ni^{2+} affinity and gel filtration fraction(s). The broad range ladder was provided by ThermoFisher and reference molecular masses are displayed in kDa. Labels are as follows: **P**: 10x diluted resuspended pellet (50mM Tris-HCl pH 7.1, 300 mM NaCl) post cell lysis; **FT**: 10x diluted flow through from the nickel column after loading; **L**: 10x diluted sample which was loaded onto the nickel column; **IW**: 10x diluted AKTA initial wash with 50 mM Tris-HCl pH 7.1, 300 mM NaCl; Lane **5**: 10x diluted combined fractions from the nickel column AKTA run as determined from chromatographs; Lane **6**: 20x diluted purified and combined fractions after gel filtration.

Crystals were optimised in 48-well plate format using 800 nL plus 800 nL (1:1) sized drops. The best crystals were derived from drops containing 0.2 M $(NH_4)_2SO_4$, 0.1 M Bis-Tris pH 5.5 and 25% (w/v) PEG 3350 with 10% (v/v) methylpentanediol 0.1 M Bis-Tris pH 5.5 and 25% (w/v) PEG 3350 with 10% (v/v) methylpentanediol (MPD). Crystals were fished and flash-cooled with liquid nitrogen with no other cryoprotectant.

For MATOUAmDH2 purified from pETYBSLIC-3C

MATOUAmDH2 was concentrated to 10 mg mL^{-1} was mixed with 10 mM $NADP^+$ and 10 mM cyclohexylamine, after which the protein solution was incubated on ice for at least 30 min. Following screening against commercial screens using the Mosquito robot (TTP LabTech) in 96 well plates, the conditions were then optimised further in 48-well and 96-well plate formats (ratios as above). The best hits were obtained from 96-well optimisations in drops containing 0.2 M $MgCl_2$, 0.1 M Bis-Tris pH 6.5 and 25%

(w/v) PEG 3350. Crystals were fished from initial crystal screens and were flash-cooled in liquid nitrogen with no additional cryoprotectant added. and their diffraction was tested using an in-house X-ray generator with a rotating anode (Rigaku). Diffraction patterns were visualised using Advx (v.1.9.14) imaging and the best sent off to the Diamond Light Source, Oxford, UK on various beamlines.

5. Data Collection and Refinement

Data were collected on beamlines I03 and I04 at the Diamond Light Source and were processed and integrated using XDS[2] and scaled using SCALA[3] within the Xia2[4] processing system. Data collection statistics can be found in **Table S2.** Crystals of MATOUAmDH2 from the pET22b construct were obtained in space group $P2_12_12_1$ with four molecules in the asymmetric unit constituting two dimers. The structure was solved with MOLREP[5] using the structure of the amine dehydrogenase from *Cystobacter fuscus* (33% sequence identity, PDB code 6IAU)[6] as the molecular replacement model. The structure was solved using iterative cycles of the programmes COOT[7] and REFMAC5.[8] After building of the protein backbone, side chains and water molecules, residual density was present in the omit map in all four active sites. This could be modelled as $NADP^+$ in each case. Crystals of MATOUAmDH2 from the pETYSBLIC3C construct were obtained in the $P3_21$ space group with one molecule in the asymmetric unit. This structure was solved using the MATOUAmDH2 structure as a model and built and refined as for the other structure. In this case the omit maps revealed clear density in the active site that could be modelled as $NADP^+$ and also the amination product cyclohexylamine. The Ramachandran plot for the $NADP^+$ complex revealed 98.4%, 1.3% and 0.3% of residues in highly preferred, preferred and outlier regions respectively. The corresponding figures for the $NADP^+$-CHA complex were 98.7%, 1.0% and 0.3%. Refinement statistics for the structures can be found in **Table S2**. Coordinates and structure factor files have been deposited in the Protein Data Bank (PDB) for the MATOUAmDH2 in complex with $NADP^+$ and with $NADP^+$ and cyclohexylamine with the accession codes **7ZBO** and **7R09** respectively.

Table S2. Data collection and refinement statistics for MATOUAmDH2 in complex with $NADP^+$ and cyclohexylamine (CHA). Numbers in brackets refer to data for the highest resolution shell.

	**MATOUAmDH2 in complex with $NADP^+$**	**MATOUAmDH2 in complex with $NADP^+$ and CHA**
Beamline	Diamond I04	Diamond I03
Wavelength (Å)	0.97950	0.97625
Resolution (Å)	64.21-2.32 (2.37-2.32)	55.40-2.08 (2.13-2.08)
Space Group	$P2_12_12_1$	$P3_21$
Unit cell (Å)	a = 91.10 b = 112.19; c = 153.15<br>α = β = γ = 90°	a = 93.51 b = 93.51; c = 75.79<br>α = β = 90° γ = 120°
No. of molecules in the asymmetric unit	4	1
Unique reflections	68673 (4580)	23365 (1768)
Completeness (%)	100.0 (100.0)	99.9 (99.9)
R_{merge} (%)	0.26 (1.37)	0.07 (1.28)
$R_{p.i.m}$.	0.14 (0.74)	0.02 (0.41)
Multiplicity	8.4 (8.3)	20.2 (20.7)
$\langle I/\sigma(I) \rangle$	5.8 (1.6)	24.4 (2.7)
Overall *B* from Wilson plot ($Å_2$)	27	48
$CC_{1/2}$	0.99 (0.68)	1.00 (0.94)
R_{cryst}/ R_{free} (%)	24.0/28.8	23.3/29.8
r.m.s.d 1-2 bonds (Å)	0.01	0.007
r.m.s.d 1-3 angles (°)	1.63	1.42
Avge main chain B ($Å^2$)	39	58
Avge side chain B ($Å^2$)	39	61
Avge water B ($Å^2$)	26	57
Avge $NADP^+$ B ($Å^2$)	38	54
Avge CHA B ($Å^2$)	-	30

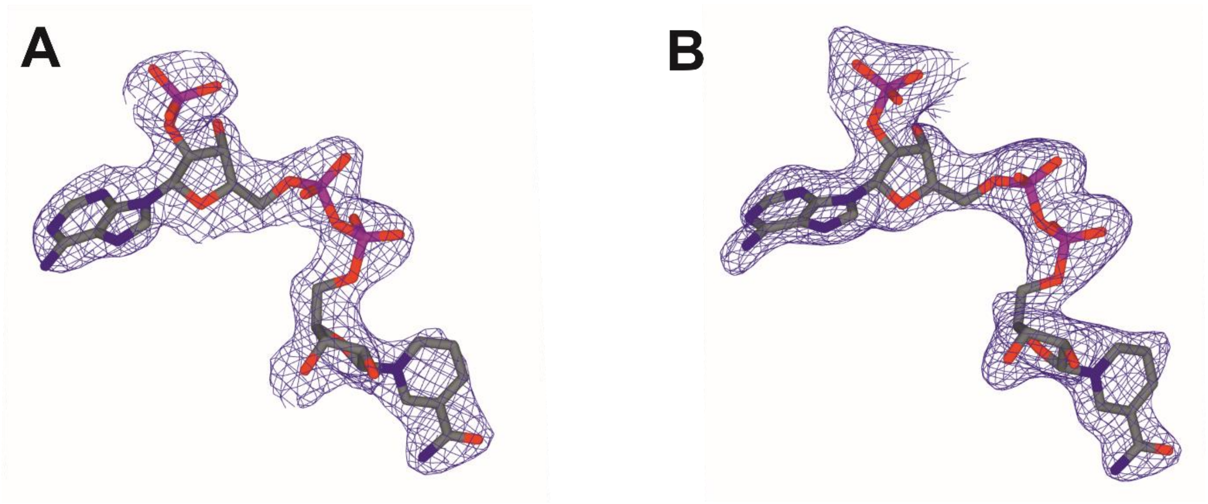

Figure S3. Omit maps for cofactor NADP$^+$ for **A:** the NADP$^+$ complex and **B**: the NADP$^+$-cyclohexylamine complex. Electron density corresponds to the omit (*F*o – *F*c) map at a level of 3σ observed prior to ligand building and refinement. The cofactor atoms from the final structures have been added for clarity.

6. Site-Directed Mutagenesis

Mutants of MATOUAmDH2 were created using the pETYSBLIC-3C construct as the template and primers that were designed with alanine mutations at sites F143, L144, L169, L180, M215 and T312. The primers are listed in **Table S3**.

Table S3. PCR primers for MATOUAmDH2 active site mutants.

Mutation	Primers	
**F143A**	Forward	TCTGATCGGCGGTGGTGCTCTGGACGGTG
	Reverse	TCACAGCAAGCACCGTCCAGAGCACCACCG
**L144A**	Forward	CGGCGGTGGTTTCGCTGACGGTGCTTGCTGT
	Reverse	CAGCAAGCACCGTCAGCGAAACCACCGCCGATC
**L169A**	Forward	AAGTTAGATGGGGGTGCTCAGTACAACGTTG
	Reverse	TCAACGTTGTACTGAGCACCCCCATCT
**L180A**	Forward	CGGTCAGGTAGCTGCTATCGCCCAC
	Reverse	TGGGCGATAGCAGCTACCTGACCGT
**M215A**	Forward	TACGTATACAACGCTAACGACTGGTTCGC
	Reverse	GCGAACCAGTCGTTAGCGTTGTATACGTAT
**T312A**	Forward	GAAAAACCCGCCGGCTCCGGCGATGA
	Reverse	GGTCATCGCCGGAGCCGGCGGGTTTTT

MATOUAmDH2 muteins were expressed and purified as for the WT enzyme in both the pET22b and pETYSBLIC-3C constructs, described in protocols above.

7. Kinetic Measurements

The protocol closely followed the one describe by Caparco and co-workers.[1] The activity of WT MATOUAmDH2 and mutants was evaluated using UV spectrophotometry by measuring substrate-stimulated NADPH oxidation at a range of cyclohexanone concentrations. The total volume of the reaction in the cuvette was 1 mL made up to the final volume with 2 M ammonium formate buffer at pH 8.0. The machine was zeroed using 1 mL of the same buffer. Cyclohexanone from stock solutions in DMSO were added to give concentrations of 0.25, 0.5, 0.75, 1, 2, 2.5, 5, 10 and 20 mM. Following addition of cyclohexanone to the buffer, the cuvette was incubated at 25ºC for 3 min and then 3 µL of a stock solution of NADPH was added to a final concentration of 0.3 mM and incubated again. 0.25 mg mL^{-1} of L180A/M215A/T312A, or 0.20 mg mL^{-1} in the case of MATOUAmDH2 pETYSBLIC-3C, were added to initiate each reaction and the cuvette contents were briefly mixed. F143A, L144A and L169A were all unstable at 25ºC and precipitated immediately on addition to the components in the cuvette. The reactions were monitored at 340 nm at a temperature of 25ºC, scanning for between 4-10 min. based on the observed plateau time. The final normalised, velocity values, calculated from an average of three runs for each enzyme, were plotted against concentration of cyclohexanone (in mM) and are shown in **Figure S4**.

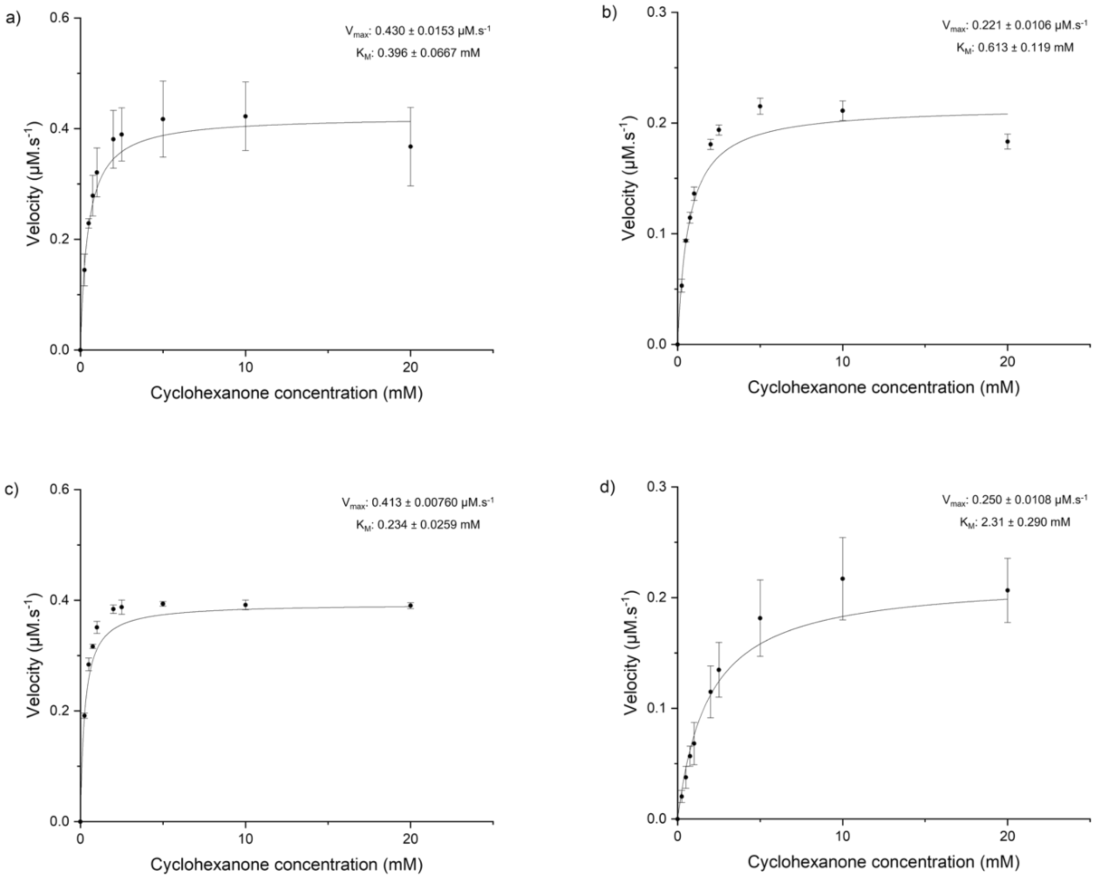

Figure S4: Kinetic plots for MATOUAmDH pETYSBLIC-3C WT, L180A, M215A and T312A. Activity was measured using UV-vis spectroscopy to monitor NADPH oxidation at 340 nm using a range of cyclohexanone concentrations. Michaelis Menten, non-linear curve fits, and standard deviation (y-axis) error bars are displayed along with V_{max} and K_m values for each variant. **a)** MATOUAmDH2 pETYSBLIC-3C WT NADPH:cyclohexanone; **b)** L180A:NADPH:cyclohexanone; **c)** M215A:NADPH: cyclohexanone; **d)** T312A:NADPH:cyclohexanone.

8. Biotransformations

The progress of amination reactions catalyzed by MATOUAmDH2 was monitored by gas chromatography (GC). Substrate and product standards were made up by adding 20 μL of ketone or amine to 580 μL ethyl acetate. Methylamine-HCl and cyclohexanone stock solutions were made up in water and DMSO respectively. DMSO concentration was kept to a constant final concentration of 1% in the total reaction mixtures. All reactions were incubated at 25ºC with shaking at 150 r.p.m. Reaction

conditions where ammonia was the amine donor contained the following in a 3 mL total reaction volume: 10 mM cyclohexanone, 12 mM glucose (1.2 equiv.), 3 U mL^{-1} (9 U) glucose dehydrogenase (G-DH), 0.5 mM NADPH with 1 mg mL^{-1} of WT MATOUAmDH2 pETYSBLIC-3C or mutants L180A, M215A or T312A, made up to 3 mL total volume with 2 M ammonium formate buffer pH 8.0. Reactions with methylamine as the amine donor contained the following in a 3 mL total reaction volume: 10 mM cyclohexanone, 12 mM glucose (1.2 equiv.), 3 U mL^{-1} (9 U) G-DH, 0.5 mM NADPH, 1 mg.mL^{-1} of WT MATOUAmDH2 pETYSBLIC-3C or mutants L180A, M215A or T312A, and 250 mM methylamine (25 equiv.) made up to 3 mL with 200 mM Tris-HCl buffer pH 8.5. Aliquots of 200 µL were taken every 1 h between t = 0-8 h and then t = 24 h, with t =0 time points being taken directly after the addition of enzyme. Aliquots were quenched with 20 µL of 10 M NaOH and then extracted with 600 µL ethyl acetate after which the organic layer was dried using $MgSO_4$ and then analysed using GC.

Samples were analysed by GC-FID using an Agilent HP-6890 GC system equipped with a Flame Ionization Detector (FID). 1 µL samples were loaded onto an Aligent HP-5ms Ultra Inert column (30 m x 250 µm x 0.25 µm) using a split ratio of 1:50 (for both ammonia and methylamine reactions). The initial oven temperature was 80ºC, following which a ramp of 15ºC min^{-1} was applied until a maximum temperature of 200ºC. Retention times for cyclohexanone, cyclohexylamine and *N*-methyl cyclohexylamine were 1.88, 1.66 and 2.05 min respectively. Peaks were processed by the data analysis interface of Aligent 7890B to give the peak areas used in conversion calculations . Each reaction for each variant was performed three times and the means and standard deviation error bars were calculated and plotted using OriginPro® (OriginLab, v.2021).

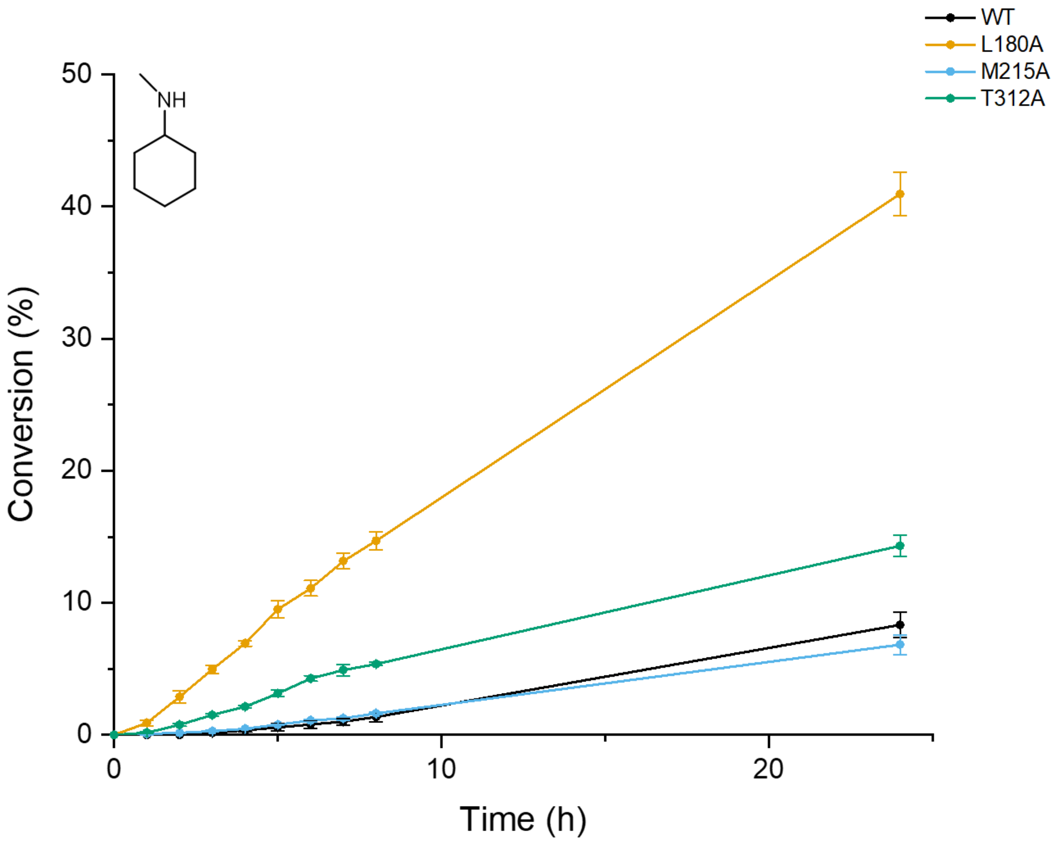

Figure S5. Biotransformation of cyclohexanone by MATOUAmDH2 and mutants using methylamine as the amine donor.

9. Modelling

Automated docking was performed using AUTODOCK VINA 1.1.2.[9] The monomer structure of MATOUAmDH2 from the $P3_21$ dataset was prepared using AUTODOCK utility scripts. Coordinates for iminium intermediate **16** were prepared using ACEDRG[10] in the ccp4 suite.The active site of MATOUAmDH2 was contained in a grid of 32 x 32 x 32 Å respectively with 0.375 Å spacing, centred around the catalytic centre which was generated using AutoGrid in the AUTODOCK Tools interface. The number of runs for genetic algorithm was set to 10 and the rest of the docking parameters were set to default parameters. The dockings were performed by VINA, therefore the posed dockings were below 2 Å rmsd. The results generated by VINA were visualised in AUTODOCK Tools 1.5.6 where the ligand conformations were assessed upon lowest VINA energy, and yielded the result in **Figure 6** as the top pose.

10. References

[1] A.A. Caparco, E. Pelletier, J.-L. Petit, A. Jouenne, B.R. Bommarius, V. de Berardinis, J.A. Champion, A.S. Bommarius and C. Vergne-Vaxelaire, *Adv. Synth. Catal.*, **2020**, *362*, 2427-2436.

[2] W. Kabsch, *Acta Crystallogr. Sect. D. Biol. Crystallogr.* **2010**, *66*, 125-132.

[3] P. Evans, *Acta Crystallogr. Sect. D. Biol. Crystallogr.* **2006**, *62*, 72-82.

[4] G. Winter, *J. Appl. Cryst*., **2010**, *3*, 186-190.

[5] A. Vagin and A. Teplyakov,. *J. Appl. Crystallogr.* **1997**, *30*, 1022-1025.

[6] O. Mayol, K. Bastard, L. Beloti, A. Frese, J.P. Turkenburg, J.-L. Petit, A. Mariage, A. Debard, V. Pellouin, A. Perret, V. de Berardinis, A. Zaparucha, G. Grogan and C. Vergne-Vaxelaire, *Nat. Catal.*, **2019**, *2*, 324-333.

[7] P. Emsley and K. Cowtan, *Acta Crystallogr., Sect D: Biol. Crystallogr.* **2004**, *60*, 2126-2132.

[8] G.N. Murshudov, A.A. Vagin and E.J. Dodson, *Acta. Crystallogr. Sect. D. Biol. Crystallogr.*, **1997**, *53*, 240-255.

[9] O. Trott and A.J. Olson, *J. Comp. Chem.*, **2010**, *31*, 455-461.

[10] F. Long, R.A. Nicholls, P. Emsley, S. Gražulis, A. Merkys, A. Vaitkus and G.N. Murshudov, *Acta Crystallogr., Sect D: Biol. Crystallogr.*, **2017**, *73*, 112-122.
